# Fungal co-inoculation enhances drought tolerance and soil functioning during cyanobacterial biological soil crust development

**DOI:** 10.3389/fmicb.2026.1947519

**Published:** 2026-09-09

**Authors:** Xiangjun Zhou, Yanghui Wu, Jinyang Han, Jingjing Chen, Naiwen Chen, Lanzhou Chen

**Affiliations:** 1 Huangshi Key Laboratory of Prevention and Control of Soil Pollution, College of Urban and Environmental Sciences, Hubei Normal University, Huangshi, China; 2 Hubei Key Laboratory of Biomass-Resources Chemistry and Environmental Biotechnology, School of Resource and Environmental Sciences, Wuhan University, Wuhan, China

**Keywords:** biological soil crusts (BSCs), cyanobacteria–fungal consortium, drought tolerance, exometabolomics, extracellular polymeric substances

## Abstract

**Background:**

Soil moisture availability strongly constrains the establishment of cyanobacteria-based biological soil crusts (BSCs). This study investigated whether fungal co-inoculation could improve drought tolerance during biocrust development.

**Methods:**

Cyanobacterial crusts (C) and cyanobacterial-fungal composite crusts (CF) were cultivated for 60 days under five soil-moisture levels (0–20%) using sand from the Hobq Desert, China. Chlorophyll a, chlorophyll fluorescence, soil nutrients, enzyme activities, EPS fractions, and GC-MS-based exometabolomics were measured.

**Results:**

CF crusts maintained higher chlorophyll a content and photosynthetic activity (Fv/Fm) under drought, particularly at 5% soil moisture. Fungal co-inoculation increased soil C and N contents, enhanced sucrase, urease, and phosphatase activities, and markedly promoted extracellular polymeric substances, especially tightly bound EPS. Exometabolomic analysis revealed greater metabolic differentiation across the moisture gradient in composite crusts, with higher diversity of carbohydrate metabolites. Under equivalent drought conditions, composite crusts accumulated higher levels of drought-responsive carbohydrates (fructose, glucose, maltose, mannobiose, trehalose), while TCA-cycle intermediates generally decreased with increasing drought severity.

**Conclusions:**

Fungal co-inoculation enhances drought resilience of engineered BSCs through enhanced extracellular matrix development and drought-responsive changes in extracellular carbohydrate profiles, providing a promising microbial consortium for improving biocrust establishment in water-limited drylands.

## 1 Introduction

Desertification is intensifying under climate change, land overuse, and unsustainable land management, threatening ecosystem stability across drylands worldwide (Burrell et al., 2020; Tariq et al., 2024). Conventional restoration measures, including afforestation and engineering-based soil stabilization, often exhibit limited effectiveness in arid environments because plant establishment and soil development are constrained by chronic water scarcity. Biological soil crusts (BSCs), complex assemblages of cyanobacteria, algae, fungi, lichens, and associated microorganisms inhabiting the soil surface, have therefore emerged as an important nature-based solution for dryland restoration (Antoninka et al., 2020; Phillips et al., 2022). By stabilizing soil particles, enhancing water retention, and promoting carbon and nutrient cycling, BSCs can improve ecosystem functioning and increase resistance to erosion in degraded landscapes (Weber et al., 2022; Gufwan et al., 2025). However, water availability remains the primary environmental factor controlling the establishment, persistence, and ecological functioning of biocrusts, often determining the success or failure of restoration efforts in arid and semi-arid ecosystems (Palanački Malešević et al., 2024; Young et al., 2024).

Among the various artificial biocrust technologies, cyanobacterial inoculation has received particular attention because cyanobacteria are pioneer colonizers capable of initiating crust formation and contributing photosynthetically derived carbon and extracellular polymeric substances (EPS) (Chamizo et al., 2018). Nevertheless, the field performance of cyanobacteria-based inoculants remains highly variable (Cubeckova et al., 2003; Román et al., 2018). Although cyanobacterial crusts can develop rapidly under favorable laboratory conditions, newly established crusts are frequently exposed to prolonged desiccation before stable extracellular matrices are formed. Under severe drought, cyanobacteria often exhibit reduced photosynthetic activity, impaired metabolic exchange, and weakened EPS production, resulting in poor crust establishment and persistence (Román et al., 2021; Wu et al., 2025). Consequently, improving drought tolerance has become a critical prerequisite for translating cyanobacterial inoculation from controlled environments to large-scale dryland restoration. Recent field-based restoration studies have shown that the establishment and persistence of inoculated biocrusts remain challenging under harsh and variable environmental conditions, highlighting the need for strategies that enhance microbial establishment and stress resistance (Alameda-Martín et al., 2024; Jech et al., 2025).

One promising strategy is to construct biocrusts as functional microbial consortia rather than relying on cyanobacteria alone (Li et al., 2026). Fungi are particularly attractive partners because they can simultaneously provide structural and biochemical functions during crust development. Through extensive hyphal networks, fungi physically bridge soil particles and enhance aggregate formation, while abundant secretion of extracellular polymers contributes to water retention and surface stabilization (Angulo et al., 2024). In addition, fungi participate actively in organic matter transformation and nutrient cycling (Yu et al., 2024), potentially creating a more favorable microenvironment for cyanobacterial growth. Recent studies have demonstrated that cyanobacteria–fungi co-inoculation can accelerate crust formation and improve soil fertility compared with single-species inoculation (Zhou et al., 2024). More mature natural biocrusts, including lichen- and fungus-associated crusts, also exhibit stronger hydraulic regulation and greater resistance to environmental stress than early cyanobacterial crusts (Shi et al., 2023). These observations suggest that fungal partners may substantially enhance drought tolerance, but the underlying mechanisms remain poorly understood.

Understanding fungal-mediated drought resistance requires examination of both extracellular matrix formation and metabolic adaptation. EPS constitutes the primary structural component of developing biocrusts and plays essential roles in water retention, soil aggregation, and protection against desiccation (Adessi et al., 2018; Costa et al., 2018; Mugnai et al., 2018). Importantly, EPS is not functionally homogeneous. Tightly bound EPS (TB-EPS), which is closely associated with microbial cells and soil particles, contributes disproportionately to soil stabilization and water-holding capacity compared with more loosely associated EPS fractions (Rossi et al., 2018; Chamizo et al., 2020). However, the response of individual EPS fractions to fungal inoculation under drought conditions remains largely unexplored. In parallel, microorganisms commonly respond to water limitation through metabolic responses, including the accumulation of compatible solutes such as sugars, sugar alcohols, and organic acids that contribute to osmotic adjustment and cellular protection (Garcia et al., 2025; Sharma et al., 2025; Gahlot, 2026). Such metabolites may contribute to osmotic adjustment and cellular protection and may also provide substrates for extracellular polymer biosynthesis (Malik et al., 2020; Romaní et al., 2025). Nevertheless, whether fungal inoculation coordinates extracellular polymer production and drought-responsive carbon metabolism has received little attention. In particular, the potential coupling between EPS fraction dynamics and carbohydrate accumulation remains unresolved, limiting the rational design of drought-resistant microbial consortia for biocrust restoration.

Based on these considerations, we hypothesized that fungal inoculation would alter drought-responsive extracellular metabolic profiles, particularly carbohydrate accumulation, while promoting both extracellular polymer production and compatible-solute accumulation, thereby enhancing photosynthetic persistence and lowering the minimum soil-moisture threshold required for biocrust development. To test this hypothesis, we established microcosms using moving sand collected from the Hobq Desert and compared cyanobacterial crusts inoculated with *Phormidium tenue* alone with composite crusts co-inoculated with *P. tenue* and the high-EPS-producing fungal strain Zh2. Both crust systems were cultivated for 60 days under a gradient of soil moisture levels (0%, 5%, 10%, 15%, and 20%). We quantified crust development and photosynthetic performance using chlorophyll *a* and chlorophyll fluorescence measurements, determined EPS fractions, evaluated soil carbon, nitrogen, and enzyme activities, and characterized extracellular metabolites using GC–MS-based exometabolomics. By integrating physiological, biochemical, and metabolomic responses, this study aims to elucidate how fungal–cyanobacterial cooperation enhances drought tolerance and to provide a mechanistic basis for the development of more resilient engineered biocrusts for dryland restoration.

## 2 Materials and methods

### 2.1 Soil collection

Surface sand was collected from mobile sandy dunes in the Hobq Desert, Dalateqi County, Inner Mongolia, China (44°21′N; 109°50′E). The Hobq Desert is a hyper plateau at an elevation of 1,230 m and characterized by extensively shifting sandy dunes in an arid to semi-arid climate. The average annual temperature is 7.4 °C, and the prevailing wind direction is WNW, with an average annual speed of 2.7 m/s. Sand samples were taken from the top 10 cm of the surface layer. The sand was air-dried, sieved through a 2 mm mesh, and used as the soil substrate for inoculation. Its bulk density, pH, and electrical conductivity were measured as 1.17 ± 0.10 g/cm^3^, 7.91 ± 0.15, and 72.6 ± 2.4 μS/cm, respectively.

### 2.2 Cultivation of biocrusts under different drought conditions

Based on the results of our previous study (Zhou et al., 2024), *Phormidium tenue* and the fungus Zh2 were simultaneously and evenly inoculated onto 60 g of moving sand to establish cyanobacterial-fungal composite crusts (CF). The inoculation density was 1 ± 0.15 μg Chl a cm^−2^ for *P. tenue* and 1 × 10^7^ CFU cm^−2^ for the fungal strain Zh2, respectively. The fungal strain Zh2 was previously isolated isolated from BSCs as a high extracellular polymeric substance (EPS)-producing strain and was identified as *Exophiala oligosperma* based on morphological characteristics and ITS rRNA gene sequencing. The obtained sequence was deposited in the NCBI database under accession number MH973233. As a control, cyanobacterial crusts (C) were prepared by inoculating *P. tenue* alone at the same cyanobacterial density (1 ± 0.15 μg Chl a cm^−2^). Based on the soil moisture range in the Hobq Desert, which varies from 3 to 18% (Wei et al., 2008), five soil moisture levels (0%, 5%, 10%, 15%, and 20%) were established. The 0% moisture treatment represented an extreme desiccation condition without external water addition, whereas the other moisture treatments were maintained at their designated gravimetric water contents by daily supplementation of sterile water to compensate for evaporation losses. The 0% and 20% treatments were included to represent extreme drought and relatively high moisture scenarios, respectively. Both CF and C were subjected to these five soil moisture treatments, with five replicates per treatment. The C groups were designated C1–C5, corresponding to soil water contents of 0%, 5%, 10%, 15%, and 20%, respectively, while the CF groups were designated CF1–CF5, following the same soil water content levels.

During the cultivation period, microcosms were weighed daily and sterile water was added to restore the target gravimetric water content in all treatments except the 0% moisture treatment. The cultures were incubated continuously at 25 °C in a lighted growth chamber with 20% relative humidity at a light intensity of 50 μEm^−2^ s^−1^ for 60 days. Samples were collected every 7 days, 5 h after water addition, for the extraction of chlorophyll *a*. At the end of the cultivation period, the photosynthetic activity of the crusts and soil nutrient levels were measured.

### 2.3 Chlorophyll *a* and chlorophyll fluorescence measurements

Then, chlorophyll *a* was extracted from 1.0 g of the soil samples using 5 mL of ethanol at 80 °C for 5 min. The samples were incubated at 4 °C for 8 h and then centrifuged at 3,500 × *g* for 15 min. The extraction was repeated twice to obtain a full pigment recovery. The pigment-containing supernatant was measured at 655 nm and 750 nm using a spectrophotometer (UV-1700 PharmaSpec, Japan). Chlorophyll *a* concentration was calculated by the formula reported by Mugnai et al. (2018).

The photosynthetic activity of the biocrust was measured as follows: after dark adaptation for 15 min, the biocrust was assessed using a portable chlorophyll fluorometer (Handy PEA). The light intensity was set to 3,000 μmol·m^−2^·s^−1^, and the fluorescence signal was recorded for 1 s.

### 2.4 Soil nutrient contents and enzyme activities

Air-dried biocrust samples were gently crushed and passed through a 2-mm sieve prior to analysis. Total carbon (C) and total nitrogen (N) contents were determined from 0.2 g of crust material wrapped in tin capsules using an elemental analyzer (Vario Macro Cube, Elementar, Germany). Soil catalase activity was determined by the potassium permanganate titration method, sucrase activity by the 3,5-dinitrosalicylic acid (DNS) colorimetric method, urease activity by the sodium phenol–sodium hypochlorite colorimetric method, and phosphatase activity by the disodium phenyl phosphate colorimetric method.

### 2.5 Extracellular polymeric substances (EPS) extraction and quantification

EPS were sequentially extracted from biocrust samples as loosely bound EPS (LB-EPS), tightly bound EPS (TB-EPS), and glycocalyx EPS (G-EPS) according to Rossi et al. (2018). LB-EPS was recovered by water extraction, TB-EPS by treatment with 0.1 M Na_2_EDTA, and G-EPS by hot-water extraction at 80 °C. The polysaccharide content of each fraction was determined using the phenol–sulfuric acid method with glucose as the calibration standard. Total EPS was calculated as the sum of the three fractions and expressed on a dry-weight basis.

### 2.6 Exometabolomic profiling of biocrusts

Non-targeted extracellular metabolite profiling was conducted using a method based on the exometabolomics approach established by Swenson et al. (2015, 2018). A 5.0 g portion of air-dried biocrust was gently crushed and kept on ice. Then, 30 mL of pre-chilled sterile Grade-1 water was added, and the mixture was shaken at 200 rpm for 1 h, followed by centrifugation at 3,220 × *g* for 15 min. The supernatant was filtered through a 0.45 μm aqueous-phase membrane, and 1.5 mL of the filtrate was transferred into a 2 mL microcentrifuge tube. Subsequently, 100 μL methanol containing 5 μg of the internal standard 2-amino-3-bromo-5-methylbenzoic acid (ABMBA; CAS 13091-43-5) was added. The samples were then freeze-dried for 6–8 h. To monitor instrument performance and data reproducibility during the analytical run, a pooled quality control (QC) sample was prepared by combining equal volumes of all sample extracts. QC samples were injected at regular intervals (every 10 injections) throughout the sequence. Metabolite features with a relative standard deviation (RSD) > 30% in the QC samples were excluded from subsequent statistical analyses to ensure data reliability.

The dried extract was reconstituted in 200 μL methanol, vortexed, sonicated for 5 min, and filtered through a 0.22 μm organic-phase membrane to obtain 100 μL of extract for derivatization. The derivatization procedure followed Kind et al. (2009). First, 10 μL of methoxyamine hydrochloride solution (40 mg/mL, dissolved in 99.99% pyridine) was added, and the mixture was incubated at 30 °C with shaking for 90 min. Then, 90 μL N-methyl-N-(trimethylsilyl) trifluoroacetamide (MSTFA) containing 1% trimethylchlorosilane (TMCS) was added and incubated at 37 °C with shaking for 90 min. After filtration through a 0.22 μm organic-phase filter, samples were analyzed using gas chromatography-mass spectrometry (GC-MS; Shimadzu, Japan).

For GC-MS analysis, high-purity helium gas (99.999%) was used as the carrier gas at a flow rate of 1.0 mL/min. The injection volume was 1 μL in splitless mode. The column temperature program was: initial temperature 35 °C for 5 min; ramp at 5 °C/min to 180 °C and hold for 1 min; then ramp at 10 °C/min to 315 °C and hold for 3 min. The solvent cut-off time was set at 12.5 min. The ion source temperature was 225 °C and the ionization energy was 70 eV. Mass spectra were acquired in the range of 10–600 m/z, and metabolites were identified by comparison with the WILEY7 and NIST11 standard libraries, and only compounds with a similarity index (SI) > 75 were retained for subsequent analyses.

### 2.7 Statistical analyses

Five biological replicates were initially prepared for each treatment. However, one replicate was excluded due to technical variation during GC–MS analysis and quality assessment, resulting in four biological replicates being used for subsequent exometabolomic analyses. Metabolite peak areas were normalized to the internal standard (ABMBA) prior to statistical analyses. Features with missing values >70% across all samples were removed. For the remaining features, missing values were imputed using the *k*-nearest neighbors (KNN) algorithm (*k* = 10) implemented in MetaboAnalyst (Developed by Xia Lab, McGill University, Montreal, QC, Canada. Available online: https://www.metaboanalyst.ca). The normalized metabolite data were mean-centered and subsequently Pareto-scaled by dividing each centered variable by the square root of its standard deviation. PLS-DA was performed separately for the combined cyanobacterial and composite crusts, composite crusts, and cyanobacterial crusts, using two latent components. Because the number of measured metabolites exceeded the number of biological replicates, model performance was evaluated by cross-validation and a 200-permutation test to assess potential overfitting. *R*^2^*X*, *R*^2^*Y*, and *Q*^2^ values were calculated to characterize the explanatory and predictive performance of each model.Hierarchical clustering analysis (HCA), and biomarker identification using MetaboAnalyst and R software (version 3.5.1) (R Foundation for Statistical Computing, Vienna, Austria).

All data were expressed as mean ± standard deviation (SD). The effects of crust type, soil moisture level, and their interaction on physiological parameters, EPS fractions, and soil biochemical properties were evaluated using two-way analysis of variance (ANOVA). When significant interactions were detected, one-way ANOVA followed by Tukey's honestly significant difference (HSD) test was performed to compare among all treatment combinations. Statistical analyses were performed at *P* < 0.05 using IBM SPSS Statistics 20. Graphs were generated using OriginPro 2025 (OriginLab Corporation, Northampton, MA, USA).

## 3 Results

### 3.1 Dynamic changes of chlorophyll *a* content at different soil moisture

**Figure 1:**
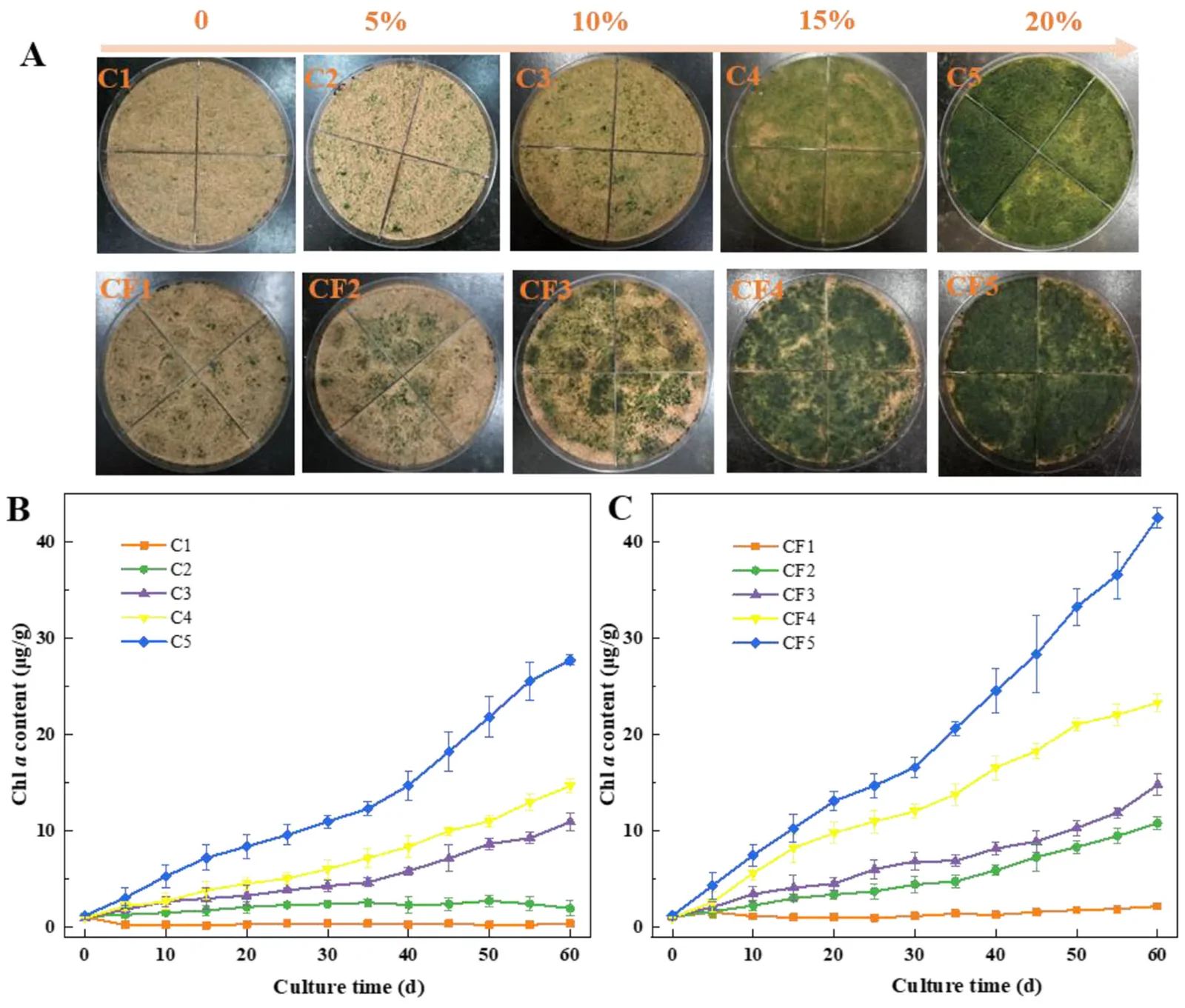
Effects of soil moisture on the development and chlorophyll *a* accumulation of cyanobacterial crusts (C) and cyanobacterial–fungal composite crusts (CF). **(A)** Representative photographs of crust development after 60 days under five soil moisture levels (0%, 5%, 10%, 15%, and 20%). Temporal changes in chlorophyll *a* content in **(B)** cyanobacterial crusts and **(C)** composite crusts during cultivation. Data are presented as mean ± SD (*n* = 5).

Biocrust development increased with soil moisture in both C and CF after 60 days of cultivation (Figure 1A). Surface greening was limited under 0% and 5% soil moisture but became more pronounced at 15% and 20%. At comparable moisture levels, CF crusts generally showed greater surface colonization than C crusts.

Chlorophyll *a* content increased over time in both crust types, with the accumulation rate strongly dependent on soil moisture (Figures 1B, C). Under 0% soil moisture, chlorophyll *a* remained nearly unchanged. At 5% soil moisture, chlorophyll *a* content reached only about 1.93 μg g^−1^ in C crusts after 60 days, whereas CF crusts accumulated approximately 10.77 μg g^−1^. Increasing soil moisture from 10 to 20% markedly enhanced chlorophyll *a* accumulation in both crust types. The highest values were observed at 20% soil moisture, reaching approximately 22.7 μg g^−1^ in C crusts and 42.5 μg g^−1^ in CF crusts. The significant crust type × soil moisture interaction for chlorophyll *a* accumulation (*F* = 42.93, *P* < 0.001) indicates that the effect of fungal co-inoculation on biomass development depended strongly on water availability (Supplementary Table S2).

### 3.2 Photosynthetic responses under different soil moisture levels

**Figure 2:**
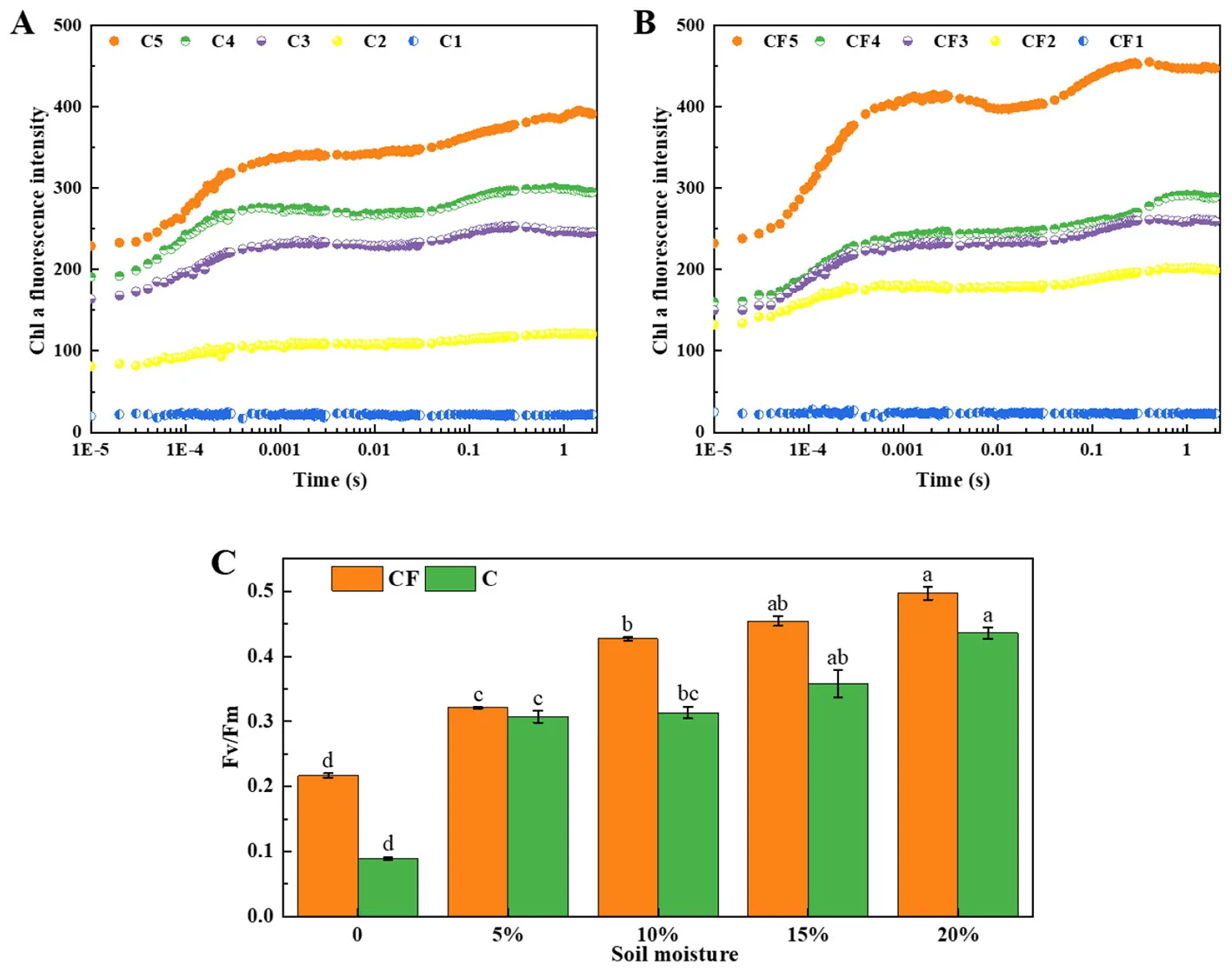
Effects of soil moisture on chlorophyll fluorescence kinetics and photosynthetic activity of cyanobacterial crusts (C) and cyanobacterial–fungal composite crusts (CF). **(A)** OJIP chlorophyll fluorescence induction curves of C crusts cultivated for 60 days under different soil moisture levels. **(B)** OJIP chlorophyll fluorescence induction curves of CF crusts. **(C)** Maximum photochemical efficiency of photosystem II (*F*_v_/*F*_m_) in the two crust systems under varying soil moisture conditions. Values represent mean ± SD (*n* = 5). Different lowercase letters denote significant differences among soil moisture treatments within each crust type (*P* < 0.05, one-way ANOVA with Tukey's HSD *post hoc* test).

Representative OJIP chlorophyll fluorescence transients measured after 60 days of cultivation are shown in Figures 2A, B. In both crust systems, fluorescence intensity increased with increasing soil moisture. Under moderate and high soil moisture conditions (10%−20%), distinct OJIP phases were observed, indicating active photosynthetic electron transport. However, drought markedly altered fluorescence kinetics. In cyanobacterial crusts, the OJIP transient became progressively flattened as soil moisture decreased and was nearly absent at 0% and 5% soil moisture. In contrast, composite crusts retained discernible fluorescence transients at 5% soil moisture, although the overall fluorescence intensity was reduced compared with wetter treatments.

The maximum quantum yield of photosystem II (PSII; *F*_v_/*F*_m_) increased significantly with increasing soil moisture in both crust systems (Figure 2C). After 60 days of cultivation, *F*_v_/*F*_m_ values ranged from approximately 0.09 to 0.44 in cyanobacterial crusts and from 0.22 to 0.50 in composite crusts. At all moisture levels, composite crusts exhibited higher *F*_v_/*F*_m_ values than cyanobacterial crusts. The difference was particularly pronounced under severe drought conditions. At 0% soil moisture, *F*_v_/*F*_m_ declined sharply in both crust systems, but composite crusts still maintained values more than twofold higher than those of cyanobacterial crusts. A significant crust type × soil moisture interaction was also observed for *F*_v_/*F*_m_ (*F* = 23.88, *P* < 0.001), indicating that the beneficial effect of fungal co-inoculation on PSII photochemical performance varied with soil moisture availability (Supplementary Table S2).

### 3.3 Soil C and N contents and enzyme activities under different soil moisture levels

**Figure 3:**
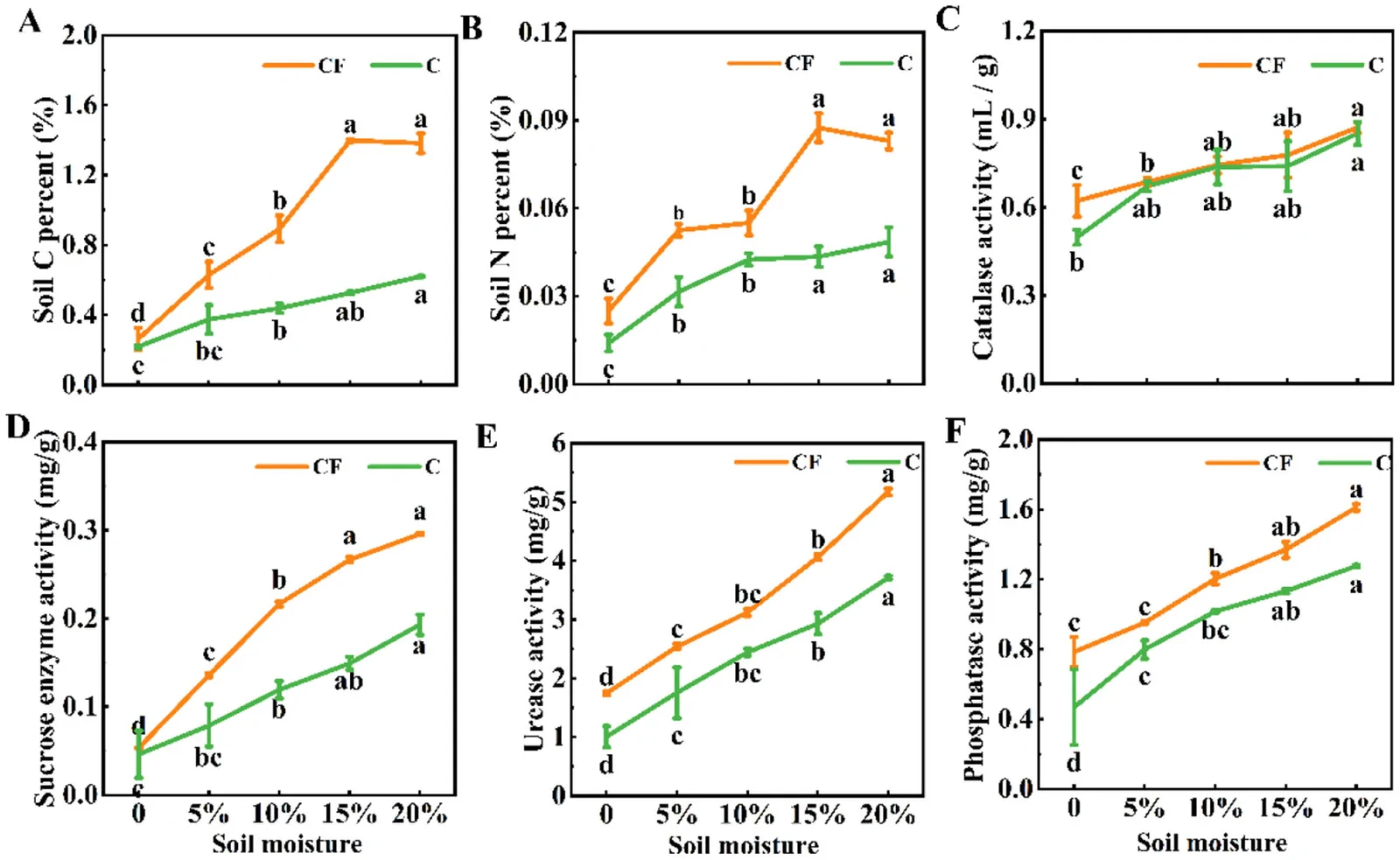
Effects of soil moisture and fungal co-inoculation on soil C and N contents and enzyme activities in biocrusts after 60 days of cultivation. **(A)** Total carbon (C); **(B)** total nitrogen (N); **(C)** catalase activity; **(D)** sucrase activity; **(E)** urease activity; and **(F)** phosphatase activity. CF, cyanobacterial–fungal composite crusts; C, cyanobacterial crusts. Values represent mean ± SD (*n* = 5). Different lowercase letters denote significant differences among soil moisture treatments within each crust type (*P* < 0.05, one-way ANOVA with Tukey's HSD *post hoc* test).

Soil carbon and nitrogen contents increased progressively with increasing soil moisture in both C and CF crusts (Figures 3A, B). Across all moisture treatments, CF crusts contained higher concentrations of both C and N than C crusts. The differences became more pronounced at intermediate and high moisture levels (10%−20%). At 20% soil moisture, soil C and N contents in CF crusts reached approximately 1.5% and 0.10%, respectively, exceeding those measured in the corresponding cyanobacterial crusts.

The activities of soil enzymes also varied substantially with moisture availability (Figures 3C–F). Catalase activity increased with increasing soil moisture in both crust systems but showed only minor differences between CF and C crusts (Figure 3C). In contrast, sucrase, urease, and phosphatase activities were consistently higher in CF crusts than in C crusts across most moisture treatments (Figures 3D–F). The largest differences were observed for urease and phosphatase activities under moderate to high soil moisture conditions. For example, phosphatase activity in CF crusts at 20% soil moisture was approximately 1.5-fold higher than that in cyanobacterial crusts.

### 3.4 EPS contents under different soil moisture levels

**Figure 4:**
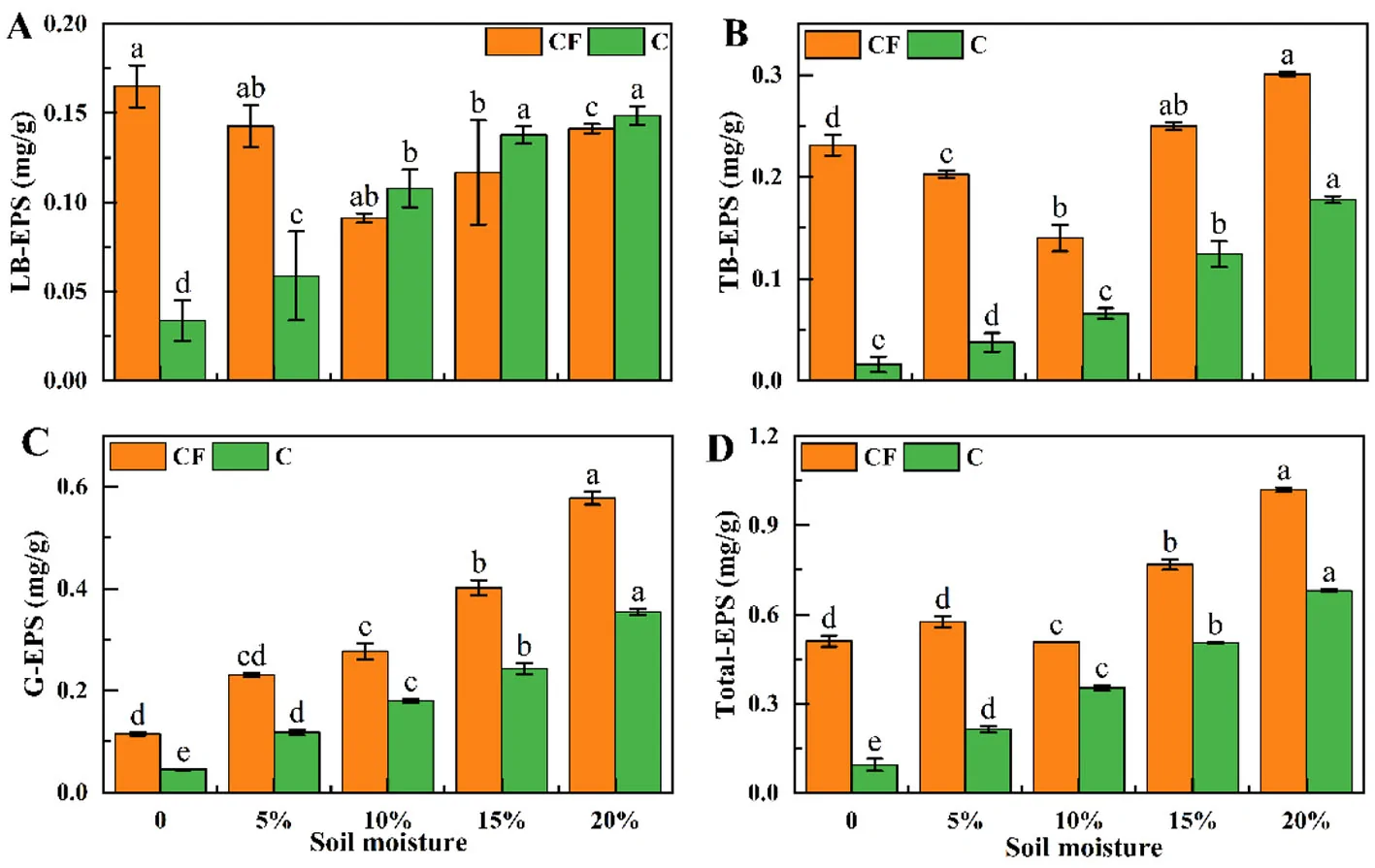
Effects of soil moisture and fungal co-inoculation on EPS fractions in biocrusts after 60 days of cultivation. **(A)** Loosely bound EPS (LB-EPS); **(B)** tightly bound EPS (TB-EPS); **(C)** glycocalyx EPS (G-EPS); and **(D)** total EPS. Values represent mean ± SD (*n* = 5). Different lowercase letters denote significant differences among soil moisture treatments within each crust type (*P* < 0.05, one-way ANOVA with Tukey's HSD *post hoc* test).

The contents of LB-EPS, TB-EPS, G-EPS, and total EPS varied substantially with soil moisture and crust type after 60 days of cultivation (Figure 4). In general, CF crusts accumulated greater amounts of EPS than C crusts, although the magnitude of this difference differed among EPS fractions. LB-EPS showed contrasting responses to soil moisture in the two crust systems (Figure 4A). In composite crusts, LB-EPS content was highest under severe drought (0%−5% soil moisture) and gradually declined with increasing soil moisture, whereas cyanobacterial crusts exhibited a progressive increase in LB-EPS content along the moisture gradient. Consequently, the difference between crust types was most pronounced under drought conditions and diminished at higher soil moisture levels. Among the three EPS fractions, TB-EPS showed the strongest response to fungal inoculation and represented the dominant contributor to total EPS accumulation in composite crusts (Figure 4B). Across all moisture treatments, TB-EPS contents in composite crusts were consistently higher than those in cyanobacterial crusts. Under severe drought (0%−5% soil moisture), TB-EPS accumulation in composite crusts was approximately 5–20 times greater than that in cyanobacterial crusts. Although TB-EPS contents increased in both crust types with increasing soil moisture, the advantage of composite crusts remained evident throughout the experiment.

G-EPS content increased progressively with soil moisture in both crust systems (Figure 4C). Composite crusts consistently contained higher G-EPS concentrations than cyanobacterial crusts, with the largest differences observed at intermediate and high soil moisture levels (10%−20%). The combined effects of these EPS fractions resulted in substantially greater total EPS accumulation in composite crusts than in cyanobacterial crusts (Figure 4D). Total EPS increased with increasing soil moisture in both crust types, reaching maximum values at 20% soil moisture. Nevertheless, composite crusts maintained markedly higher total EPS contents across all moisture treatments. Among the three EPS fractions, TB-EPS contributed the largest proportion of the EPS pool and represented the fraction most strongly enhanced by fungal inoculation.

### 3.5 Exometabolomic responses to drought stress

**Figure 5:**
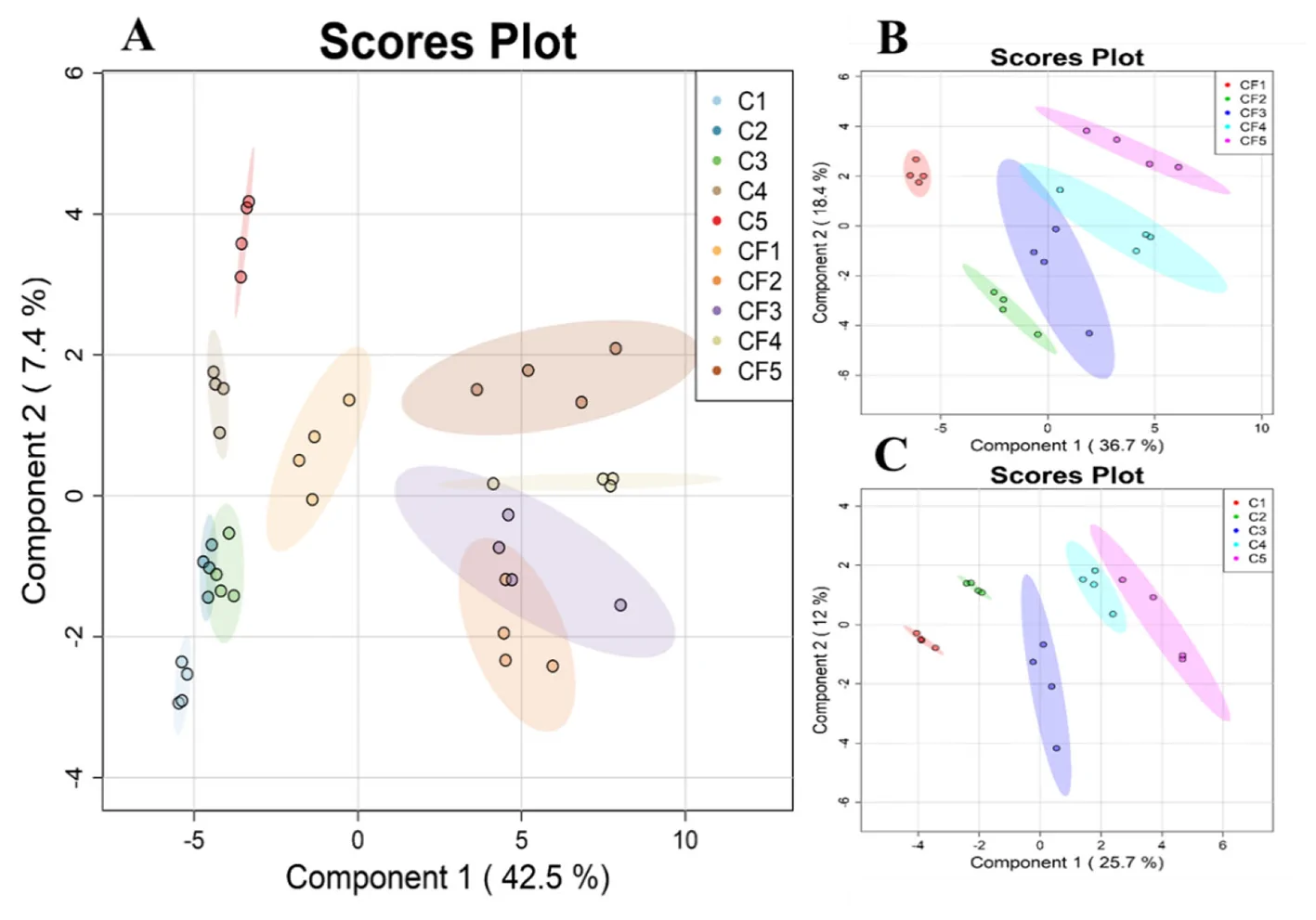
PLS-DA score plots of extracellular metabolites in biocrusts subjected to different soil moisture treatments. **(A)** Combined analysis of cyanobacterial crusts (C) and cyanobacterial–fungal composite crusts (CF). **(B)** Composite crusts (CF). **(C)** Cyanobacterial crusts (C). Numbers in parentheses indicate the variance explained by each component.

To visualize overall metabolic responses to drought, PLS-DA was performed using Pareto-scaled metabolite abundance data (Figure 5). Clear separation among moisture treatments was observed, with the first two components explaining 42.5% and 7.4% of the total variance, respectively (Figure 5A). Compared with C crusts, CF crusts occupied a broader ordination space, indicating stronger metabolic differentiation across the soil-moisture gradient. When analyzed separately, CF1 formed a distinct cluster clearly separated from the remaining treatments, whereas CF3–CF5 showed partial overlap (Figure 5B). In contrast, substantial overlap was observed between C1 and C2, while greater separation occurred among C3–C5 (Figure 5C). These results indicate that extracellular metabolite profiles of composite crusts were more responsive to changes in soil moisture than those of cyanobacterial crusts. The PLS-DA models showed acceptable explanatory and predictive performance. For the combined C and CF dataset, the two-component model explained 52.7% of the variance in the metabolite matrix (*R*^2^*X* = 0.527), with *R*^2^*Y* and *Q*^2^ values of 0.214 and 0.204, respectively. For the CF and C datasets, *R*^2^*X*/*R*^2^*Y*/*Q*^2^ values were 0.568/0.470/0.431 and 0.540/0.485/0.470, respectively. The 200-permutation tests supported the non-random nature of the observed models and provided no evidence of substantial overfitting (Supplementary Table S3).

A total of 94 extracellular metabolites were detected across all samples, including 68 metabolites in composite crusts and 64 in cyanobacterial crusts, with 36 shared metabolites. Hierarchical clustering analysis (Supplementary Figures S1, S2) generally supported the PLS-DA results. Notably, composite crusts contained a substantially larger repertoire of sugar-related metabolites than cyanobacterial crusts (30 vs. 12 metabolites). Several carbohydrates, including fructose, allose, talose, trehalose, maltose, and β-gentiobiose, accumulated preferentially under moderate drought (5% soil moisture), whereas only a limited number of drought-responsive sugars were detected in cyanobacterial crusts. These results suggest that fungal inoculation enhanced drought-associated metabolic plasticity, particularly in carbohydrate metabolism.

### 3.6 Identification of key metabolites

**Figure 6:**
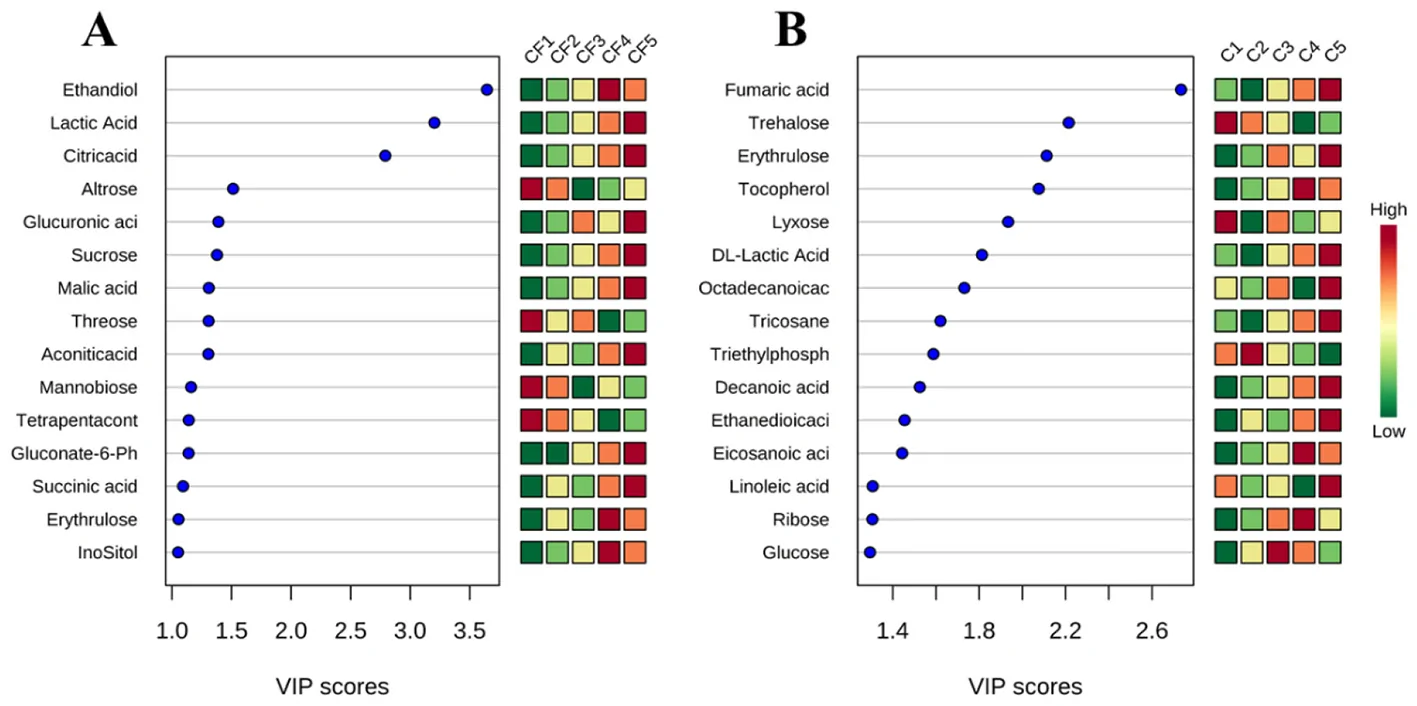
Drought-responsive metabolites identified using VIP scores and significance testing (*P* < 0.05). **(A)** Cyanobacterial–fungal composite crusts (CF). **(B)** Cyanobacterial crusts (C). Metabolites are ranked according to their VIP scores. Colored boxes indicate relative metabolite abundance across soil-moisture treatments, from low (green) to high (red).

To identify metabolites contributing most strongly to drought-associated metabolic variation, metabolites with VIP > 1.0 and *P* < 0.05 were selected as key discriminatory compounds (Figure 6). Fifteen metabolites were identified in both composite and cyanobacterial crusts. In the composite crusts, the key metabolites comprised five sugars, six organic acids, and several other compounds (Figure 6A). Most organic acids, including citrate, malate, lactate, and gluconate-6-phosphate, increased progressively with increasing soil moisture. In contrast, several carbohydrates, including altrose, threose, and mannobiose, accumulated preferentially under drought conditions and reached their highest relative abundance at low soil moisture. These metabolites contributed strongly to the separation of drought-stressed and well-watered composite crusts in the PLS-DA model. In cyanobacterial crusts, 15 drought-responsive metabolites were also identified (Figure 6B), including five sugars and eight organic acids. Several metabolites associated with favorable growth conditions, such as fumarate and lactate, increased with soil moisture. Conversely, trehalose and lyxose accumulated under severe drought stress and represented major contributors to the metabolic differentiation of low-moisture treatments.

Notably, composite crusts exhibited a broader diversity of drought-responsive carbohydrates than cyanobacterial crusts. Several sugars enriched under drought in the composite crusts were not detected among the major discriminatory metabolites of cyanobacterial crusts, suggesting that fungal inoculation enhanced carbohydrate-based metabolic responses to water limitation.

### 3.7 Differential extracellular metabolites between composite and cyanobacterial crusts under severe drought

**Figure 7:**
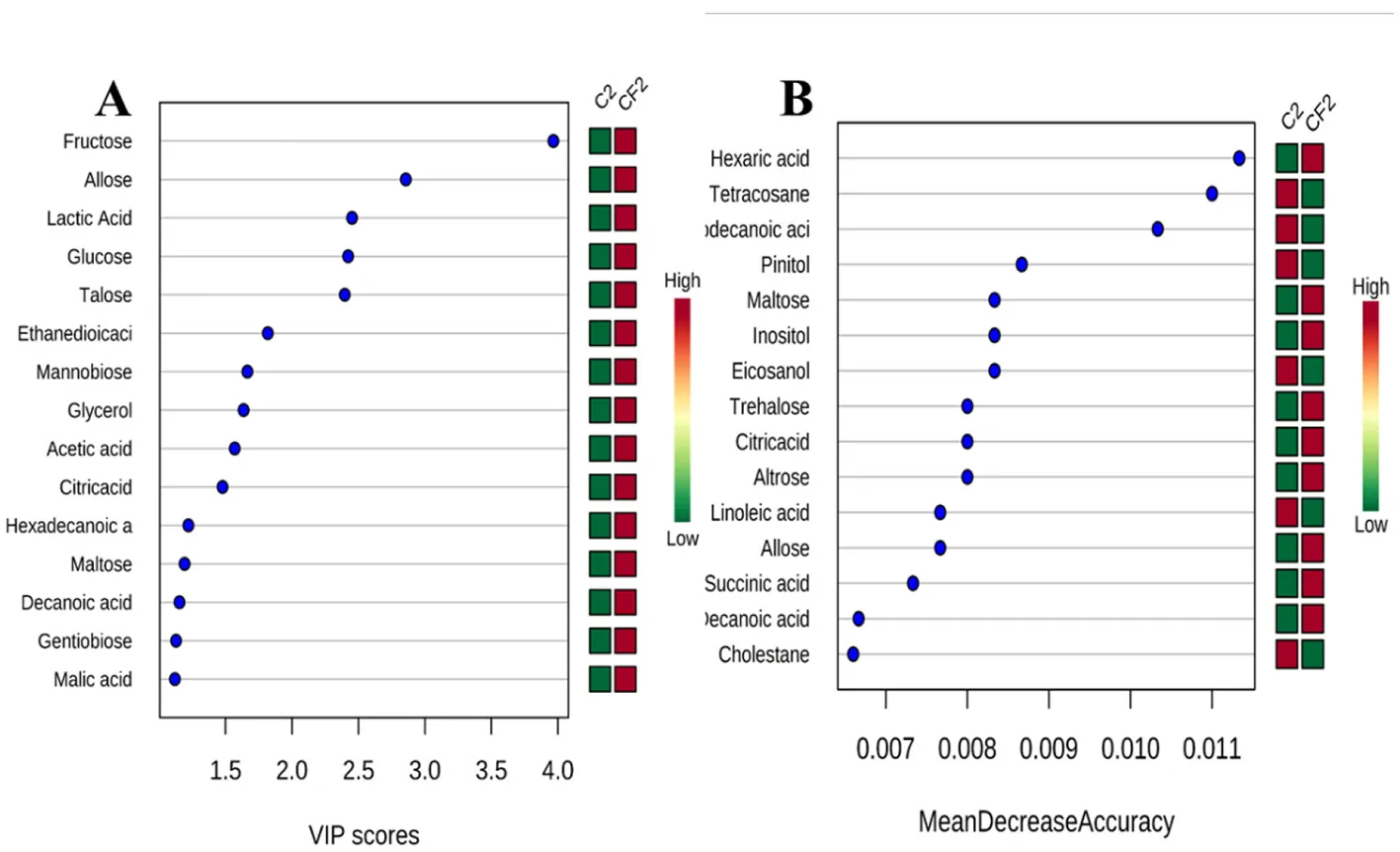
Key metabolites distinguishing cyanobacterial–fungal composite crusts (CF2) from cyanobacterial crusts (C2) under 5% soil moisture. **(A)** Metabolites ranked according to PLS-DA variable importance in projection (VIP) scores. **(B)** Metabolites ranked according to Random Forest Mean Decrease Accuracy values. Colored boxes indicate relative metabolite abundance in each crust type, with red representing higher abundance and green representing lower abundance.

To identify metabolic differences associated with fungal inoculation under equivalent drought conditions, extracellular metabolites in CF2 and C2 cultivated at 5% soil moisture were compared using PLS-DA and Random Forest analyses (Figure 7). PLS-DA identified 15 discriminatory metabolites with VIP values >1.0 (Figure 7A). Among these metabolites, several carbohydrates, including fructose, allose, glucose, talose, mannobiose, maltose, and gentiobiose, accumulated to substantially higher levels in CF2 than in C2. In contrast, only a limited number of non-carbohydrate metabolites contributed strongly to the separation between the two crust types. The higher VIP scores observed for multiple sugars indicate that carbohydrate metabolism represented the primary source of metabolic differentiation under drought conditions.

Random Forest analysis produced a similar pattern (Figure 7B). Of the 15 most important metabolites ranked by Mean Decrease Accuracy, nine compounds, including maltose, trehalose, inositol, allose, citric acid, succinic acid, and hexanoic acid, were enriched in CF2 relative to C2. Conversely, several metabolites, including tetracosane, pinitol, eicosanol, and linoleic acid, showed higher abundance in the cyanobacterial crusts.

## 4 Discussion

Water availability is widely recognized as the dominant factor controlling the establishment, persistence, and ecological functioning of BSCs in arid and semi-arid ecosystems. The present study demonstrates that fungal co-inoculation substantially enhanced the drought tolerance of *Phormidium tenue*-based biocrusts by simultaneously promoting physiological stability, extracellular matrix development, nutrient retention, and drought-associated metabolic responses under water-limited conditions.

The enhanced drought resistance of the composite crusts was first reflected in their growth and photosynthetic responses. Chlorophyll *a* accumulation increased with soil moisture in both crust systems, but fungal inoculation markedly improved crust development under water-limited conditions. At 5% soil moisture, cyanobacterial crusts showed only minimal biomass accumulation throughout the cultivation period, whereas composite crusts continued to grow and accumulated more than five times higher chlorophyll *a* concentration after 60 days (Figures 1B, C). Similar differences were observed in chlorophyll fluorescence responses. Under severe drought, OJIP fluorescence transients in cyanobacterial crusts became nearly flattened, indicating substantial impairment of PSII photochemistry and electron transport. In contrast, composite crusts retained discernible fluorescence transients and significantly higher Fv/Fm values under the same moisture conditions (Figures 2A–C). These results suggest that fungal inoculation effectively lowered the minimum moisture threshold required to maintain photosynthetic activity and biomass production, a key determinant of biocrust establishment and recovery following hydration events (Rajeev et al., 2013; Xu et al., 2021). Such an effect is particularly important during the early stages of artificial biocrust establishment, when insufficient water availability frequently limits cyanobacterial colonization and survival under field conditions (Alameda-Martín et al., 2024; Jech et al., 2023; Kimura and Okuro, 2025).

The improved physiological performance of the composite crusts was closely associated with changes in EPS. Although fungal inoculation increased total EPS accumulation, the most pronounced response occurred in TB-EPS, which was consistently enriched in composite crusts across all soil-moisture treatments (Figure 4B). Across the entire moisture gradient, TB-EPS concentrations were consistently higher in composite crusts than in cyanobacterial crusts, with differences becoming particularly pronounced under severe drought. Under 0%−5% soil moisture, TB-EPS accumulation in composite crusts was approximately one order of magnitude greater than that in cyanobacterial crusts (Figure 4B). This distinction is mechanistically important because different EPS fractions perform different ecological functions. LB-EPS is relatively mobile and can be readily released into the surrounding environment, whereas TB-EPS is closely associated with microbial cells and soil particles and contributes disproportionately to soil aggregation, water retention, and structural stability (Rossi et al., 2018; Chowaniec et al., 2025). Furthermore, cyanobacterial and fungal EPS can create hydrated microenvironments surrounding microbial cells, thereby buffering desiccation stress and prolonging physiological activity following hydration events (Romaní et al., 2025). The significant crust type × soil moisture interaction for TB-EPS (*F* = 42.90, *P* < 0.001) provides statistical support for the moisture-dependent enhancement of TB-EPS associated with fungal co-inoculation. A greater accumulation of TB-EPS may increase the water-binding capacity and structural stability of the extracellular matrix, potentially contributing to the maintenance of a more hydrated microenvironment around cyanobacterial filaments (Chamizo et al., 2020; Kidron, 2021). However, these hydrological effects were not directly measured in the present study and therefore remain a plausible mechanism rather than a demonstrated outcome. The close correspondence among TB-EPS accumulation, chlorophyll *a* production, and chlorophyll fluorescence responses (Figures 1, 2, 4) supports a potential role of extracellular matrix development in the enhanced drought performance of the composite crusts.

Changes in soil biochemical properties further indicate that fungal inoculation improved the functional environment of developing crusts. Across the moisture gradient, composite crusts consistently contained higher carbon and nitrogen contents than cyanobacterial crusts (Figures 3A, B). These findings are consistent with our previous work showing that fungal hyphal networks promote carbon sequestration and nutrient retention during biocrust development (Zhou et al., 2024). Activities of sucrase, urease, and phosphatase were also significantly higher in composite crusts than in cyanobacterial crusts (Figures 3D–F). Because these enzymes participate directly in carbon, nitrogen, and phosphorus mineralization processes, their increased activity suggests accelerated nutrient turnover and enhanced nutrient availability within the crust microenvironment (Zhu et al., 2024; Zhao et al., 2025). Improved nutrient availability may facilitate cyanobacterial recovery following repeated wetting–drying cycles and contribute to the higher biomass accumulation observed in composite crusts (Kang et al., 2024; Feuring et al., 2026). In contrast, catalase activity exhibited only minor differences between crust types (Figure 3C), indicating that enhanced antioxidant defense was unlikely to represent the primary mechanism responsible for improved drought tolerance. Neither the main effect of crust type nor the crust type × moisture interaction was significant for catalase activity (Supplementary Table S2), suggesting that fungal co-inoculation did not substantially alter catalase activity across the moisture gradient. Instead, the current evidence more strongly supports a mechanism involving enhanced resource retention and nutrient cycling.

The metabolomic analyses revealed that fungal inoculation was associated with distinct drought-responsive extracellular metabolic profiles. PLS-DA showed substantially stronger separation among moisture treatments in composite crusts than in cyanobacterial crusts, indicating greater metabolic responsiveness to changes in water availability (Figure 5). A particularly notable feature was the expansion of carbohydrate diversity. Approximately 30 sugar-related metabolites were detected in composite crusts, compared with only 12 in cyanobacterial crusts. Moreover, several carbohydrates, including fructose, allose, glucose, maltose, mannobiose, talose, and β-gentiobiose, accumulated preferentially under drought conditions and were significantly enriched in composite crusts relative to cyanobacterial crusts under equivalent drought stress (Figures 6, 7). Soluble carbohydrates are widely recognized as compatible solutes that stabilize proteins, membranes, and cellular structures during dehydration by maintaining osmotic balance and protecting macromolecular integrity (Cruz de Carvalho et al., 2015; Ozturk et al., 2021). Recent studies have emphasized that microbial drought adaptation involves not only structural modifications but also dynamic metabolic adjustments, including the accumulation of compatible solutes and stress-responsive metabolites that maintain cellular homeostasis under water limitation (Gahlot, 2026). In this context, the increased diversity and abundance of soluble carbohydrates observed in the composite crusts may represent a metabolic adaptation strategy that enhances osmotic adjustment and protects microbial cells during dehydration. Trehalose accumulation occurred in both crust systems, consistent with its established role in membrane stabilization and protection against protein denaturation under desiccation stress (Sakamoto et al., 2009; Yobi et al., 2012; Williams et al., 2015; Han et al., 2024). However, the composite crusts accumulated a much broader spectrum of drought-responsive carbohydrates than cyanobacterial crusts. The increased availability of soluble sugars may not only support osmotic adjustment during dehydration but also provide carbon sources for extracellular polymer production, thereby contributing to drought resistance.

In contrast to the accumulation of soluble carbohydrates, several intermediates of the tricarboxylic acid (TCA) cycle, including citrate, malate, succinate, and fumarate, generally declined with increasing drought severity (Figures 6, 7). These metabolites reached their highest abundance under relatively moist conditions and decreased progressively as soil moisture declined. Similar reductions in TCA-cycle intermediates have been reported in drought-stressed plants and photosynthetic microorganisms and are commonly interpreted as suppression of respiratory carbon flux under water limitation (Li et al., 2025; Sharma et al., 2025). Although metabolite abundance alone cannot directly quantify metabolic fluxes, suggesting changes in metabolic priorities under drought stress, with increased accumulation of stress-associated carbohydrates and reduced abundance of several TCA-cycle intermediates. Notably, many of the carbohydrates enriched under drought can also serve as substrates or precursors for EPS biosynthesis (Rossi and De Philippis, 2016). Therefore, the metabolomic results provide a plausible mechanistic link between carbohydrate accumulation and the preferential enhancement of TB-EPS observed in composite crusts (Figures 4, 6, 7). Together, these findings suggest that fungal inoculation was associated with enhanced accumulation of drought-responsive carbohydrates and increased TB-EPS production. These responses may contribute to osmotic adjustment and extracellular matrix development under drought conditions, although direct carbon flux measurements are required to confirm whether carbon allocation was altered. Recent multi-omics studies have demonstrated that metabolite-level responses are critical for linking microbial interactions with functional adaptation strategies under environmental stress conditions (Kumar et al., 2023). Therefore, the extracellular metabolic signatures identified in this study provide a complementary perspective for understanding how fungal–cyanobacterial interactions contribute to drought resilience during biocrust development.

The ecological significance of these findings extends beyond laboratory cultivation conditions. Soil moisture levels of 5%−15% broadly correspond to environmental conditions encountered across many desert regions of northern China. In the eastern desert regions of northeastern China and eastern Inner Mongolia, annual precipitation typically ranges from 250 to 500 mm (Yang et al., 2016), corresponding approximately to the 10%−15% soil-moisture treatments examined in this study. In contrast, annual precipitation in the Hobq Desert generally ranges from 150 to 250 mm, representing conditions closer to the 5%−10% treatments. Under these moisture conditions, cyanobacterial crusts exhibited severe physiological inhibition, whereas composite crusts maintained detectable photosynthetic activity and continued biomass accumulation (Figures 1, 2). This suggests that fungal-assisted inoculation may substantially improve establishment potential and increase resilience during the early stages of biocrust restoration under water-limited conditions. In more arid regions, such as parts of the Taklamakan Desert where annual precipitation is often below 50 mm, even composite crusts may approach their physiological limits. Nevertheless, the enhanced drought tolerance conferred by fungal inoculation suggests that engineered microbial consortia may represent a promising strategy for expanding the climatic envelope suitable for biocrust restoration and stabilization in increasingly water-limited dryland ecosystems.

However, these findings should be interpreted with consideration of the differences between controlled laboratory conditions and natural dryland environments. The present study evaluated drought responses under controlled and relatively stable soil moisture conditions, whereas desert ecosystems experience highly variable precipitation regimes characterized by episodic rainfall and repeated wetting–drying cycles. Recent studies have emphasized that hydration frequency and environmental filtering strongly influence the establishment and functioning of artificial biocrusts under field conditions (Young et al., 2025; Qiu et al., 2026). Indeed, recent field-based assessments have shown that successful establishment of inoculated biocrusts remains challenging due to complex environmental constraints, including moisture variability and habitat conditions (Jech et al., 2025), highlighting the importance of validating engineered microbial consortia under realistic environmental fluctuations. Although the enhanced photosynthetic persistence, EPS accumulation, and drought-responsive metabolic adjustments observed in composite crusts suggest improved tolerance to water limitation, their capacity to recover following natural rehydration events requires further validation. Another limitation is that the experimental design included cyanobacterial crusts and cyanobacteria–fungus composite crusts but not a fungus-only treatment. Therefore, the present results demonstrate the benefits associated with fungal co-inoculation but cannot distinguish the independent effects of fungal activity from potential synergistic interactions between the two microbial partners. Future experiments incorporating fungus-only and factorial inoculation treatments would allow the independent and interactive effects of the two partners to be quantified. In addition, laboratory conditions with relatively stable temperature and light regimes cannot fully represent desert environments characterized by temperature fluctuations, high radiation, and multiple interacting stresses. Therefore, long-term field experiments and dynamic drought–rewetting simulations are needed to evaluate the ecological performance of engineered cyanobacteria–fungus consortia under realistic scenarios.

## 5 Conclusions

Water availability strongly regulated the development and functioning of both cyanobacterial and cyanobacterial–fungal biocrusts. Compared with cyanobacterial crusts, fungal co-inoculation significantly enhanced chlorophyll *a* accumulation, photosynthetic activity, soil C and N contents, enzyme activities, and EPS production across the soil-moisture gradient, with the strongest effects occurring under severe drought (5% soil moisture). Notably, composite crusts maintained active photosynthesis and continued biomass accumulation under conditions where cyanobacterial crusts showed substantial physiological inhibition. Among the EPS fractions, TB-EPS exhibited the greatest response to cyanobacteria–fungus co-inoculation and was closely associated with improved drought performance. Exometabolomic analyses further revealed that cyanobacteria–fungus co-inoculation increased carbohydrate diversity and promoted the accumulation of drought-responsive sugars, including fructose, allose, maltose, mannobiose, and trehalose, while TCA-cycle intermediates generally declined with increasing drought severity. These results suggest that cyanobacteria–fungus co-inoculation enhanced drought tolerance through combined changes in extracellular matrix development, physiological performance, and drought-responsive metabolite accumulation, whereby increased TB-EPS production was associated with enhanced extracellular matrix development and may contribute to improved moisture buffering capacity, while carbohydrate accumulation may support osmotic adjustment under water limitation. Overall, fungal coinoculation effectively shifted the lower moisture threshold for biocrust development, enabling sustained photosynthetic activity and growth at 5% soil moisture, and thus represents a promising strategy for improving biocrust restoration in water-limited drylands.

## Data Availability

The raw data supporting the conclusions of this article will be made available by the authors, without undue reservation.
