# Supplementary material for: Fungal co-inoculation enhances drought tolerance and soil functioning during cyanobacterial biological soil crust development
Source: Front Microbiol. 2026 Sep 9;17:1947519. doi: 10.3389/fmicb.2026.1947519 (PMC13597704; doi:10.3389/fmicb.2026.1947519)
Supplement: Supplementary file 1 [file Supplementary_File_1.docx]

Supplementary Material

# 1 Supplementary Figures and Tables

## 1.1 Supplementary Figures

**Fig.S1 Heatmap and hierarchical clustering of extracellular metabolites in cyanobacterial–fungal composite biocrusts under different soil moisture treatments.** (A) Non-sugar metabolites. (B) Sugar-related metabolites, including sugars and sugar alcohols. Metabolite abundances were Pareto-scaled prior to clustering. Rows represent metabolites and columns represent soil-moisture treatments. Red and blue indicate relatively high and low metabolite abundance, respectively.

**Fig. S2. Heatmap and hierarchical clustering of extracellular metabolites in cyanobacterial biocrusts under different soil moisture treatments.** (A) Sugars and organic acids. (B) Other metabolites. Metabolite abundances were Pareto-scaled prior to clustering. Red and blue indicate relatively high and low metabolite abundance, respectively.

## 1.2 Supplementary Table

**Table S1. Identification information of extracellular metabolites detected by GC–MS analysis**

|  | SI | R.time | Chemical Formula (Derivatized) | Chemical Formula | Compound |
| --- | --- | --- | --- | --- | --- |
| M1 | 94 | 15.191 | C_8_H_22_O_2_Si_2_ | C_2_H_6_O_2_ | 1,2-Ethanediol |
| M2 | 76 | 15.78 | C_11_H_24_ | C_11_H_24_ | Undecane |
| M3 | 85 | 16.242 | C_12_H_26_ | C_12_H_26_ | 3,8-Dimethyldecane |
| M4 | 86 | 16.675 | C_13_H_34_O_3_Si_3_ | C_4_H_10_O_3_ | 1,2,3-Butanetriol |
| M5 | 87 | 16.691 | C_10_H_26_O_2_Si_2_ | C_4_H_10_O_2_ | Butanediol |
| M6 | 74 | 17.269 | C_12_H_26_O_2_Si | C_9_H_18_O_2_ | Nonanoic acid |
| M7 | 92 | 17.277 | C_9_H_22_O_3_Si_2_ | C_3_H_6_O_3_ | D-Lactic Acid |
| M8 | 95 | 18.044 | C_8_H_20_O_3_Si_2_ | C_2_H_4_O_3_ | Acetic acid |
| M9 | 94 | 19.632 | C_6_H_15_O_4_P | C_6_H_15_O_4_P | Triethyl phosphate |
| M10 | 82 | 20.177 | C_8_H_18_O_4_Si_2_ | C_2_H_2_O_4_ | Ethanedioic acid |
| M11 | 92 | 20.374 | C_9_H_27_O_4_PSi_3_ | H_3_PO_4_ | Phosphoric acid |
| M12 | 78 | 20.833 | C_10_H_24_O_3_Si_2_ | C_4_H_8_O_3_ | Isobutyric acid |
| M13 | 80 | 21.544 | C_15_H_32_O_2_Si | C_12_H_24_O_2_ | Dodecanoic acid |
| M14 | 89 | 23.832 | C_12_H_32_O_3_Si_3_ | C_3_H_8_O_3_ | Glycerol |
| M15 | 89 | 24.677 | C_16_H_34_ | C_16_H_34_ | Hexadecane |
| M16 | 82 | 25.126 | C_11_H_24_O | C_11_H_24_O | 2,4-Diethyl-1-heptanol |
| M17 | 80 | 25.358 | C_10_H_26_OSi_2_ | C_4_H_10_O | Butanol |
| M18 | 76 | 25.375 | C_10_H_22_O_4_Si_2_ | C_4_H_6_O_4_ | Succinic acid |
| M19 | 94 | 25.887 | C_12_H_30_O_4_Si_3_ | C_3_H_6_O_4_ | Glyceric acid |
| M20 | 87 | 26.182 | C_13_H_28_ | C_13_H_28_ | 5-Isobutylnonane |
| M21 | 79 | 26.555 | C_10_H_20_O_4_Si_2_ | C_4_H_4_O_4_ | Fumaric acid |
| M22 | 79 | 27.1 | C_15_H_32_ | C_15_H_32_ | Pentadecane |
| M23 | 92 | 28.893 | C_17_H_36_ | C_17_H_36_ | Heptadecane |
| M24 | 81 | 28.894 | C_17_H_36_O_2_Si | C_14_H_28_O_2_ | Myristicacid |
| M25 | 82 | 29.273 | C_14_H_35_NO_4_Si_3_ | C_4_H_8_O_4_ | L-(+)-Threose |
| M26 | 88 | 29.595 | C_16_H_41_NO_4_Si_4_ | C_3_H_6_O_4_ | L-(+)-Erythrulose |
| M27 | 92 | 30.273 | C_13_H_30_O_5_Si_3_ | C_4_H_6_O_5_ | Malic acid |
| M28 | 86 | 30.275 | C_20_H_42_ | C_20_H_42_ | Eicosane |
| M29 | 87 | 30.517 | C_14_H_30_ | C_14_H_30_ | 4,6-Dimethyldodecane |
| M30 | 86 | 30.668 | C_16_H_42_O_4_Si_4_ | C_4_H_10_O_4_ | meso-Erythritol |
| M31 | 74 | 30.978 | C_13_H_26_O_2_Si | C_10_H_18_O_2_ | 9-Decenoicacid |
| M32 | 84 | 31.092 | C_11_H_23_NO_3_Si_2_ | C_5_H_7_NO_3_ | L-Pyroglutamic acid |
| M33 | 79 | 31.614 | C_16_H_40_O_5_Si_4_ | C_4_H_8_O_5_ | L-Threonic acid |
| M34 | 82 | 32.266 | C_20_H_42_O_2_Si | C_17_H_34_O_2_ | Heptadecanoic acid |
| M35 | 78 | 32.267 | C_22_H_46_ | C_22_H_46_ | Docosane |
| M36 | 79 | 32.435 | C_12_H_25_NO_5_Si_2_ | C_5_H_6_O_5_ | Glutaric acid |
| M37 | 84 | 33.061 | C_12_H_14_O_4_ | C_12_H_14_O_4_ | Diethyl Phthalate |
| M38 | 80 | 33.3 | C_17_H_44_O_4_Si_4_ | C_5_H_12_O_4_ | pentitol |
| M39 | 85 | 34.446 | C_18_H_45_NO_5_Si_4_ | C_6_H_10_O_5_ | d-Ribose |
| M40 | 82 | 34.989 | C_21_H_52_O_6_Si_5_ | C_6_H_12_O_6_ | L-(-)-Sorbose |
| M41 | 85 | 35.35 | C_18_H_38_ | C_18_H_38_ | Heptadecane |
| M42 | 89 | 36.014 | C_20_H_52_O_5_Si_5_ | C_5_H_12_O_5_ | D-(+)-Arabitol |
| M43 | 79 | 36.3 | C_21_H_52_O_6_Si_5_ | C_6_H_12_O_6_ | D-(+)-Talofuranose |
| M44 | 80 | 36.739 | C_15_H_30_O_6_Si_3_ | C_6_H_6_O_6_ | cis-Aconitic acid |
| M45 | 73 | 36.96 | C_27_H_69_O_10_PSi_7_ | C_6_H_13_O_10_P | 6-Phospho-D-gluconate |
| M46 | 90 | 37.057 | C_21_H_52_O_6_Si_5_ | C_6_H_12_O_6_ | D-(-)-Tagatose |
| M47 | 81 | 37.132 | C_21_H_50_O_7_Si_5_ | C_6_H_10_O_7_ | 2-Keto-l-gluconic acid |
| M48 | 79 | 37.151 | C_20_H_50_O_6_Si_5_ | C_5_H_10_O_6_ | Ribonic acid |
| M49 | 90 | 37.705 | C_21_H_52_O_6_Si_5_ | C_6_H_12_O_6_ | D-(-)-Fructofuranose |
| M50 | 78 | 38.069 | C_18_H_40_O_7_Si_4_ | C_6_H_8_O_7_ | Citric acid |
| M51 | 73 | 38.409 | C_22_H_54_O_6_Si_5_ | C_7_H_14_O_6_ | D-Pinitol |
| M52 | 86 | 38.93 | C_21_H_52_O_6_Si_5_ | C_6_H_12_O_6_ | D-Fructose |
| M53 | 90 | 39.04 | C_22_H_55_NO_6_Si_5_ | C_6_H_12_O_6_ | D-Psicose |
| M54 | 90 | 39.794 | C_22_H_55_NO_6_Si_5_ | C_6_H_12_O_6_ | D-Allose |
| M55 | 89 | 39.943 | C_21_H_52_O_6_Si_5_ | C_6_H_12_O_6_ | D-(+)-Talose |
| M56 | 77 | 40.079 | C_23_H_48_O_2_Si | C_20_H_40_O_2_ | Eicosanoic acid |
| M57 | 80 | 40.171 | C_37_H_89_NO_11_Si_8_ | C_12_H_22_O_11_ | 2-.alpha.-Mannobiose |
| M58 | 83 | 40.395 | C_23_H_50_OSi | C_20_H_42_O | 3,7,11,15-tetramethylhexadecan-3-ol |
| M59 | 96 | 40.455 | C_21_H_52_O_6_Si_5_ | C_6_H_12_O_6_ | D-Glucose |
| M60 | 82 | 41.488 | C_19_H_40_O_2_Si | C_16_H_32_O_2_ | Hexadecanoic acid |
| M61 | 85 | 41.879 | C_21_H_52_O_6_Si_5_ | C_6_H_12_O_6_ | D-Altrose |
| M62 | 88 | 41.894 | C_24_H_60_O_6_Si_6_ | C_6_H_12_O_6_ | Myo-InoSitol |
| M63 | 78 | 42.053 | C_21_H_50_O_7_Si_5_ | C_6_H_10_O_7_ | arabino-Hexaric acid |
| M64 | 83 | 42.123 | C_21_H_44_ | C_21_H_44_ | Heneicosane |
| M65 | 89 | 42.257 | C_27_H_56_ | C_27_H_56_ | Heptacosane |
| M66 | 81 | 42.57 | C_25_H_52_ | C_25_H_52_ | Pentacocane |
| M67 | 80 | 42.721 | C_21_H_46_OSi | C_18_H_38_O | Octadecanol |
| M68 | 88 | 42.849 | C_24_H_50_ | C_24_H_50_ | Tetracosane |
| M69 | 86 | 43.322 | C_21_H_40_O_2_Si | C_18_H_32_O_2_ | Linoleic acid |
| M70 | 79 | 43.651 | C_21_H_44_O_2_Si | C_18_H_36_O_2_ | Octadecanoic acid |
| M71 | 75 | 43.83 | C_14_H_28_O_6_ | C_14_H_28_O_6_ | Octyl glucoside |
| M72 | 73 | 44.065 | C_29_H_62_O_4_Si_2_ | C_23_H_46_O_4_ | Glyceryl arachidate |
| M73 | 85 | 44.198 | C_27_H_66_O_8_Si_6_ | C_9_H_18_O_8_ | Galactosyl glycerol |
| M74 | 73 | 44.247 | C_31_H_56_O_2_Si | C_28_H_48_O_2_ | D-gamma-Tocopherol |
| M75 | 85 | 44.35 | C_24_H_62_O_6_Si_6_ | C_6_H_14_O_6_ | D-Sorbitol |
| M76 | 73 | 44.565 | C_29_H_50_O_2_ | C_29_H_50_O_2_ | A-Tocopherol |
| M77 | 87 | 44.723 | C_18_H_45_NO_5_Si_4_ | C_6_H_10_O_5_ | D-(-)-Lyxose |
| M78 | 83 | 44.777 | C_21_H_50_O_7_Si_5_ | C_6_H_10_O_7_ | D-Glucuronic acid |
| M79 | 85 | 44.957 | C_36_H_86_O_11_Si_8_ | C_12_H_22_O_11_ | D-(+)-Trehalose |
| M80 | 84 | 45.03 | C_20_H_52_O_5_Si_5_ | C_5_H_12_O_5_ | Xylitol |
| M81 | 95 | 45.781 | C_18_H_44_O_5_Si_4_ | C_6_H_12_O_5_ | Mannose |
| M82 | 81 | 46.036 | C_37_H_89_NO_11_Si_8_ | C_12_H_22_O_11_ | Maltose |
| M83 | 91 | 46.453 | C_54_H_110_ | C_54_H_110_ | Tetrapentacontane |
| M84 | 78 | 46.743 | C_25_H_54_O_4_Si_2_ | C_19_H_38_O_4_ | Monopalmitoyl glycerol |
| M85 | 95 | 47.083 | C_36_H_86_O_11_Si_8_ | C_12_H_22_O_11_ | Sucrose |
| M86 | 88 | 47.56 | C_37_H_89_NO_11_Si_8_ | C_12_H_22_O_11_ | D-(+)-Cellobiose |
| M87 | 86 | 47.568 | C_36_H_86_O_11_Si_8_ | C_12_H_22_O_11_ | Turanose |
| M88 | 86 | 47.784 | C_37_H_89_NO_11_Si_8_ | C_12_H_22_O_11_ | .beta.-Gentiobiose |
| M89 | 75 | 48.278 | C_27_H_58_O_4_Si_2_ | C_21_H_42_O_4_ | Monostearin |
| M90 | 87 | 48.711 | C_33_H_79_NO_10_Si_7_ | C_11_H_20_O_10_ | Palatinose |
| M91 | 75 | 48.95 | C_27_H48 | C_27_H_48_ | Cholestane |
| M92 | 92 | 49.864 | C_39_H_94_O_11_Si_9_ | C_12_H_22_O_11_ | Galactinol |
| M93 | 76 | 50.189 | C_23_H_48_ | C_23_H_48_ | 5-Butyl-5-ethylheptadecane |
| M94 | 90 | 51.032 | C_36_H_86_O_11_Si_8_ | C_12_H_22_O_11_ | 4-O-.beta.-Galactopyranosyl-D-mannopyranose |

**Table S2. Two-way ANOVA results for the effects of crust type, soil moisture, and their interaction on physiological, EPS, soil biochemical, and enzyme activity parameters.**

| Parameter | Crust type | | Soil moisture | | Interaction | |
| --- | --- | --- | --- | --- | --- | --- |
|  | F | P | F | P | F | P |
| Chl a | 486.24 | <0.001 | 1163.78 | <0.001 | 42.93 | <0.001 |
| F_v_/F_m_ | 385.78 | <0.001 | 647.86 | <0.001 | 23.88 | <0.001 |
| LB-EPS | 28.15 | <0.001 | 8.42 | 0.003 | 23.41 | <0.001 |
| TB-EPS | 1530.81 | <0.001 | 202.23 | <0.001 | 42.90 | <0.001 |
| G-EPS | 1010.00 | <0.001 | 1001.45 | <0.001 | 41.78 | <0.001 |
| Total-EPS | 2720.57 | <0.001 | 1116.16 | <0.001 | 59.19 | <0.001 |
| Soil N | 205.84 | <0.001 | 99.98 | <0.001 | 13.92 | <0.001 |
| Soil C | 431.46 | <0.001 | 154.90 | <0.001 | 45.03 | <0.001 |
| Catalase activity | 3.51 | 0.090 | 21.26 | <0.001 | 1.00 | 0.452 |
| Sucrase activity | 181.36 | <0.001 | 151.55 | <0.001 | 12.46 | <0.001 |
| Urease activity | 17.00 | 0.002 | 119.49 | <0.001 | 3.96 | 0.035 |
| Phosphatase activity | 49.36 | <0.001 | 67.07 | <0.001 | 1.03 | 0.440 |

**Table S3. Performance and permutation-based validation of PLS-DA models for exometabolomic profiles of cyanobacterial and composite biocrusts.**

| PLS-DA model | No. of samples | Components | R²X | R²Y | Q² | Permutation test (P) |
| --- | --- | --- | --- | --- | --- | --- |
| C + CF | 40 | 2 | 0.527 | 0.214 | 0.204 | 0.005 |
| CF | 20 | 2 | 0.568 | 0.470 | 0.431 | 0.005 |
| C | 29 | 2 | 0.540 | 0.485 | 0.470 | 0.005 |

**Note:** R²X and R²Y indicate the proportions of variance in X and Y explained by the PLS-DA model, respectively, whereas Q² indicates the predictive ability estimated by cross-validation. Models were constructed using two latent components after mean-centering and Pareto scaling. Potential overfitting was assessed using 200 permutation tests; *P* values indicate the proportion of permuted models with performance equal to or better than the original model.
